# Institutional conflicts of interest in health-related research — a scoping review

**DOI:** 10.1186/s41073-026-00251-w

**Published:** 2026-09-16

**Authors:** Alexander Haarmann, Charlotte M. Kugler, Hengameh Goodarzi, Alexander Pachanov, Kaethe Goossen, Elie A. Akl, Dawid Pieper

**Affiliations:** 1 Institute for Health Services and Health System Research, Faculty of Health Sciences, Brandenburg Medical School (Theodor Fontane), Rüdersdorf, Germany; 2 Center for Health Services Research, Brandenburg Medical School (Theodor Fontane), Rüdersdorf, Germany; 3 Institute for Research in Operative Medicine, Witten/Herdecke University, Cologne, Germany; 4 Department of Internal Medicine, American University of Beirut, Beirut, Lebanon; 5 Department of Health Research Methods, Evidence, and Impact (HEI), McMaster University, Hamilton, Canada

**Keywords:** Conflict of interest, Research integrity, Financial incentives, Healthcare provider, Clinical practice, Medicine, Health policy

## Abstract

**Background:**

Institutional conflicts of interest (COIs) have received less attention than individual COIs in health-related research.

**Objective:**

To explore how institutional COIs arising from affiliation with a healthcare provider have been studied or discussed in the literature on health-research involving humans.

**Methods:**

We conducted a scoping review following the JBI guidance and reported it according to PRISMA-ScR. The review included English‑ or German‑language empirical or conceptual publications that addressed institutional COIs —either independently or in conjunction with individual COI — within health‑related research involving humans conducted at institutions that deliver healthcare services. We systematically searched PubMed, Embase (Elsevier), and Google Scholar in July 2024. Two reviewers independently screened publications. One reviewer extracted data and another checked it. One author mapped and analysed included papers according to the categories of institutional and individual COIs that they addressed, which another author verified.

**Results:**

We included 48 publications published between 1998 and 2022. The majority (88%; *n* = 42/48) focused on North America, were empirical (67%; *n* = 32/48), and referred to institutions such as hospitals and clinics (58%; *n* = 28/48), or universities and academic institutions (58%; *n* = 28/48). Most institutional COIs described were linked to health-related industries (79%; *n* = 38/48). Almost all publications mentioned direct financial institutional COIs (98%; *n* = 47/48) primarily in relation to receiving gifts, endowments, or grants (94%; *n* = 45/48). One third of publications (*n* = 16/48) described indirect financial institutional COIs, most frequently through increasing services (27%; *n* = 13/48). Direct financial individual COI were represented in 73% (*n* = 35/48) of publications, and indirect financial individual COIs in 6% (*n* = 3/48). Half of the publications (52%; *n* = 25/48) illustrated non-financial COIs, particularly related to prestige and reputation (31%; *n* = 15/48), or career advancements and professional roles (23%; *n* = 11/48). All publications acknowledged that institutional COIs can have negative consequences, most frequently due to bias and distortion in the research process (94%; *n* = 45/48). The vast majority of publications (94%; *n* = 45/48) offered recommendations for managing COIs, including institutional policies (77%; *n* = 37/48).

**Conclusion:**

Direct financial COIs dominate the literature on institutional COIs, while indirect or non-financial COIs remain under-researched. Institutional COIs are consistently recognised as problematic and institutional policies are most frequently proposed as means to manage them.

**Supplementary Information:**

The online version contains supplementary material available at https://doi.org/10.1186/s41073-026-00251-w.

## Background

Publications on conflicts of interest (COIs) cover a broad range of topics including the individual interest of physicians or healthcare providers (cf. [1]), associations between guideline recommendations and COIs (cf. [2]), and a growing body of literature on regulations, the legal situation, and the way the field adheres to or circumvents these rules (cf. [3]). In general, a COI “exists when a past, current, or expected interest creates a significant risk of inappropriately influencing an individual’s judgment, decision, or action when carrying out a specific duty” [4]. However, definitions of COIs vary, as no single definition has achieved broad acceptance (cf. [5]).

COIs in the healthcare sector can introduce bias in patient treatment and research, thereby reducing the quality of patient care and undermining the trustworthiness of research and medicine in general [6].

In 2022, Akl et al. proposed a new framework for categorising COIs in health research [4]. The framework distinguishes between the “individual” interests and interests “through institutional affiliation”. Individual COIs comprise financial, intellectual (e.g. participation in studies, on guideline panels, or expression of opinion), and personal interests (e.g. beliefs or personal characteristics). Institutional COIs recognise that judgements, decisions, and actions may be biased not only by individuals’ interests, but also by the interests of the institutions these individuals are affiliated with. Institutional interests may arise when the institution that an individual belongs to has a financial or non-financial interest, the individual is aware of it and may be influenced by that interest [4]. For example, an institution may receive gifts from commercial entities, hold intellectual property rights, deliver and financially benefit from providing specific services (e.g. specialised patient therapy). Individual COIs of senior officials who act on behalf of the institution are also defined as institutional COIs. Institutional COIs are categorised as either financial or non-financial [4]. Both individual and institutional financial COIs are split into direct and indirect financial benefits. We define direct financial benefits as all measurable monetary gains, whereas indirect financial benefits encompass advantages that are not directly quantifiable. For individuals, indirect financial benefits may arise through their professional status, “when an individual is engaged in a specified activity as one’s main paid occupation". Indirect financial institutional COIs may arise when the institution “delivers and financially benefits from providing specific services.” [4].

In the published literature, institutional and individual COIs are often conflated and discussed together [7–9]. Because institutional interests often manifest themselves through the actions, decisions, and judgements of the individuals employed by an organisation, many studies do not clearly distinguish between the two concepts. As a result, descriptions of institutional COIs frequently overlap with discussions of individual COIs, which complicates attempts to investigate institutional COIs in isolation. For this reason, we focus our review on institutional COIs, but we also report the individual COIs that are presented alongside to capture the full scope of how the two are intertwined in the literature.

Prior research on COIs has focused predominantly on individual COIs, especially individual financial COIs [2, 3, 6]. Publications that address institutional COIs [7, 8, 10–12] have mainly examined institutional financial interests with direct financial benefits to the institution, thereby covering only a portion of the proposed framework by Akl and colleagues, which also includes indirect financial and non-financial interests [4].

The overall purpose of this scoping review was to understand how institutional COIs have been examined in the literature, to identify the research gaps and the lessons learnt, and — where reported—to describe the accompanying individual conflicts of interest [4]. Given the variety of elements that can constitute an institutional COI and the relevance for our own research context of health services research, we focus on healthcare providers, particularly those involved in health-related research involving humans.

### Research questions

How are COIs through institutional affiliation to a healthcare provider studied or discussed in the literature on health-related research involving humans? How do these institutional COIs relate to, differ from, or overlap with individual COIs?

## Methods

To answer these questions, we conducted a scoping review and followed the JBI guidance for Scoping Reviews [13]. For reporting, we adhered to the Preferred Reporting Items for Systematic review and Meta-Analysis extension for Scoping Reviews PRISMA (PRISMA-ScR) guidelines [14] (Additional File 1).

### Registered protocol

We recycled parts of this article and its wording from the previously published protocol [15], which we registered on the Open Science Framework, in accordance with guidance of the Text Recycling Research Project guidelines [16, 17].

### Eligibility criteria for studies

#### Inclusion criteria

##### Concept

We focused our scoping review on institutional COIs. According to Akl et al. [4], an institutional COI “relates to the institution with which the individual is affiliated but only to those interests of which the individual is aware” [4]. We also included publications that addressed both individual and institutional COIs when institutional COIs were explicitly presented. We excluded publications that did not provide a substantial contribution to institutional COI (i.e., those lacking discussion, analysis, examples, or recommendations related to institutional COIs).

##### Context

The context of interest was health-related research involving humans [18] carried out within institutions (i.e., entities that employ one or more individuals) that provide healthcare services (hereafter referred to as healthcare providers), such as hospitals, medical schools, medical centres, and ambulatory care practices. We limited our focus on healthcare providers and health-related research involving humans, because a feasibility test indicated that including broader institutional COI contexts would likely result in too many eligible full-texts [15]. We excluded publications that addressed institutional COIs outside the context of health-related research involving humans – such as those focusing on healthcare education, policy-making, healthcare provision, institutional review boards, COI management policies, publishing, animal studies, or laboratory research.

##### Types of sources of evidence

We included any type of empirical or conceptual publication published in English or German, reflecting the team’s language proficiency, provided as full-text. We considered studies supported by evidence (e.g. case reports, focus groups, interviews, data analyses) as empirical while any other type of publication (e.g. commentaries, opinion pieces, viewpoints, frameworks) as conceptual. Conference abstracts or posters were not eligible.

### Information sources

In July 2024, we searched the databases PubMed and Embase (Elsevier) for literature. This was complemented with a search of grey literature using Google Scholar (cf. [19]).

### Search strategy

The search strategies were developed by one of the authors experienced in information retrieval and management, including search filter development, and checked by another author against the Peer Review of Electronic Search Strategies (PRESS) criteria. [20]. The PubMed search strategy was formulated using terms and subject headings identified in articles retrieved through an initial database search. Next, we adapted the search strategy for the Embase (Elsevier) database. The publication date was not restricted. The full search strategies are provided in Additional File 2. Given the expected high number of publications to be included and to limit the workload to a manageable level, the protocol did not include additional approaches such as citation chasing.

All identified records were subsequently collated and uploaded using the Rayyan software for processing reviews [21].

### Selection of sources of evidence

After removing duplicates, two reviewers independently screened titles and abstracts for eligibility. We conducted a pilot test including the first 50 titles and abstracts, after which the two reviewers met to calibrate the application of the selection criteria, ensuring consistency over the screening process. We did not alter the selection criteria. Subsequently, two reviewers independently screened the remaining titles and abstracts. Conflicts were resolved through discussion involving a third reviewer if necessary.

We retrieved publications that proved potentially eligible for inclusion in full text. Again, two reviewers independently assessed full texts against the inclusion criteria. Disagreements were resolved through discussion and by involving a third reviewer when in doubt. For both screening steps, we used Rayyan as an electronic tool [21].

### Data charting process

One reviewer extracted the data using a self-designed extraction sheet (Microsoft Excel 2021), and a second reviewer checked accuracy of the extracted data (cf. [13]).

### Data items

The extracted data items included study characteristics, details on institutional COIs addressed (type of institutions, industries involved, type of COI), described consequences of COIs and recommendations on how to mitigate institutional COIs (see Additional File 3). We adjusted data items where necessary and, where meaningful, multiple categories per publication could apply.

### Critical appraisal of individual sources of evidence

We did not perform a quality appraisal.

### Synthesis of evidence

We analysed the types of benefits received by institutions and individuals that publications identified as potential COIs. For data synthesis, we applied Akl et al.’s framework [4] to categorise a large part of the data (see description of categories above), but slightly adapted some of the framework’s categories to deliver meaningful results. For the remaining data, we used an iterative, inductive approach of content analysis (cf. [13, 22]) to categorise the extracted data. Codes were refined iteratively, leading to increasingly general categories, that reflected the relative importance of each category. We calculated frequencies and percentages of all included publications with R and visualised the data using Excel 2021. For illustration of the different types of COIs, we selected examples from the included publications that clearly describe the respective type of COI. For each COI category, one author reviewed the included publications until identifying a particularly illustrative example. She then summarised the chosen example, and, if needed, complemented details from original publications for understanding. A second reviewer checked the selection of the example and the summary and all co-authors checked if they understood the summary.

### Public involvement

No public, patient, or interest holder involvement occurred in this study. We did not involve interest holders due to the “bias blind spot”, describing that due to unconscious bias people are more optimistic about their own biases than those of others [1]. Due to their own unconscious bias, we concluded that involving, for example, university officials would not add value to this scoping review. Patients were not involved because the scoping review did not directly address healthcare or patient experiences. Similarly, we decided against including members of the public as no specific knowledge or lived experience could have been expected.

### AI tools

During preparation of this work, we used ChatGPT-5.2 (OpenAI) for the following purposes: (1) summarising and rephrasing texts of included publications to support the reviewer’s understanding, and (2) generation of a primary draft for categorisation; we then revised and adapted the suggested categories. We also employed Le Chat (Mistral AI) and ChatGPT-5.2 (OpenAI) for (3) rephrasing, grammar, and language editing of manuscript paragraphs. The authors take full responsibility for the accuracy and integrity of all content in this publication.

### Deviations from the protocol

We rephrased the eligibility criteria to align them with the JBI framework; however, we made no substantive changes to the eligibility criteria.

We initially planned to extract and analyse institutional COIs exclusively. However, many included publications discussed individual and institutional COIs together, and descriptions of institutional COIs frequently overlapped with discussions of individual COIs, which complicated attempts to investigate institutional COIs in isolation. For example, many publications offered recommendations for COI management, yet, it was often unclear whether these applied solely to institutional COIs or also encompassed individual COIs. For this reason, we included publications if any institutional COI was reported as originally planned. We complemented our research questions and extracted the institutional and individual COIs that were presented to capture the full scope of how the two COI concepts are intertwined in the evidence base.

## Results

### Study inclusion

**Fig. 1:**
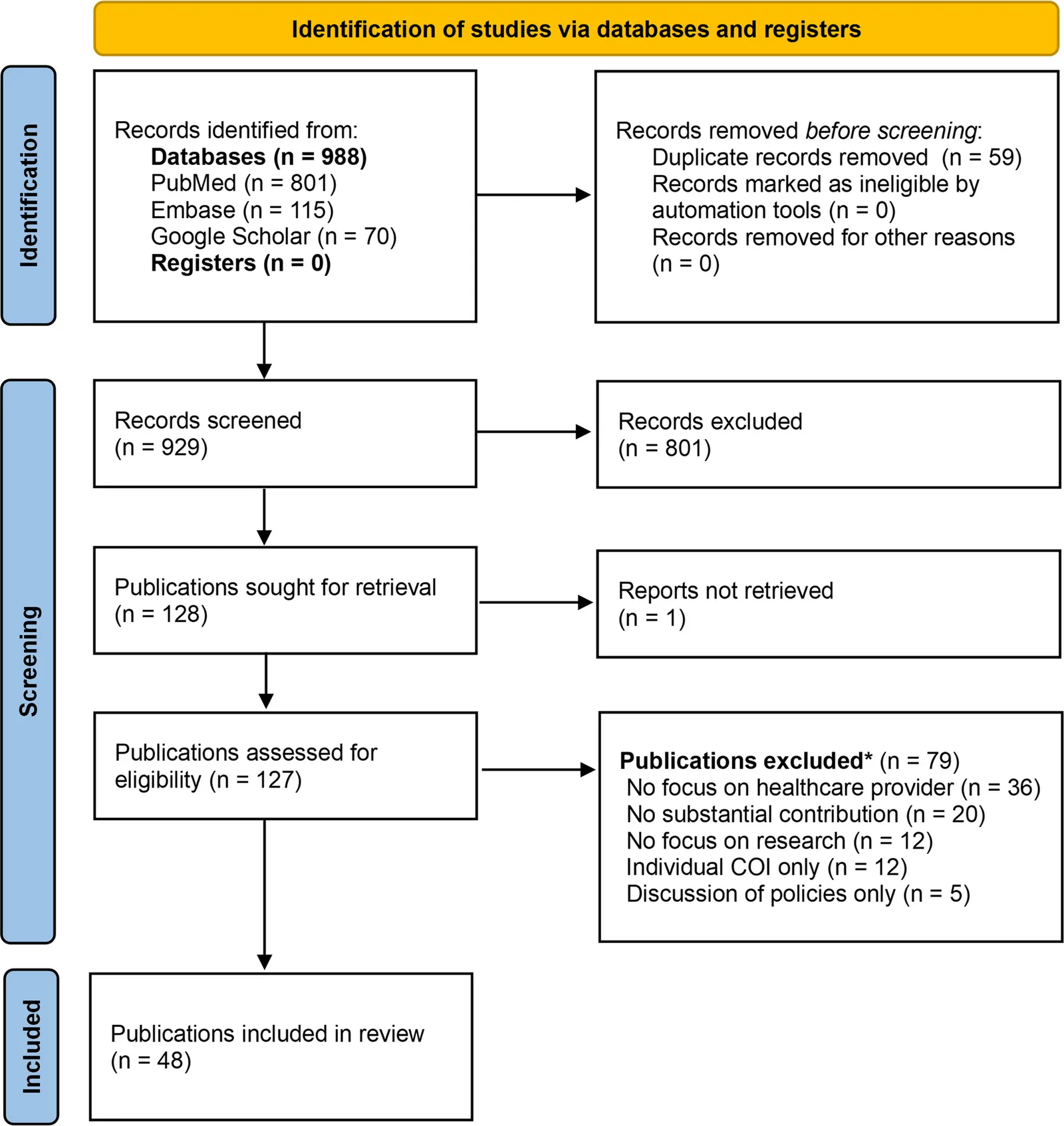
PRISMA Flowchart for the Scoping Review Process. Note: *More than one exclusion reason possible

We identified 986 records from databases and, after removing 58 duplicates, screened 930 titles and abstracts. Of these, 129 were eligible for full text screening. The main reasons for exclusion were a lack of focus on healthcare providers (n = 36 publications), or the absence of substantial contribution to the topic (n = 20, e.g. [23]). We included a total of 48 publications in this scoping review [5, 7, 9–12, 24–65] (see Fig. 1).

### Characteristics of sources of evidence

Table 1 shows characteristics of the included publications (see Additional File 4 for references). Detailed information for each included publication is provided in Additional file 3. More than two-thirds (n = 32/48) of the included publications had a strong focus on institutional COIs, as compared to a focus on individual COIs. The sample of included publications was composed of 46% (n = 22/48) of publications that addressed institutional COIs exclusively and 21% (n = 10/48) that focused on institutional COIs while individual COIs played a subordinate role. Nineteen per cent of publications (n = 9/48) equally emphasised both institutional and individual COIs and 15% (n = 7/48) had a stronger focus on individual COIs, while still addressing institutional COI (See Additional File 4).

**Table 1:** Characteristics of all included studies (n = 48)

Characteristics	Frequency (%)
	Total: 48
**Year of publication**	
Before 2000	1 (2)
2000–2004	12 (25)
2005–2009	9 (19)
2010–2014	9 (19)
2025–2019	9 (19)
2020–2022	8 (17)
Country, region*	
USA	35 (73)
Canada	7 (15)
Global	4 (8)
Australia	2 (4)
Japan	2 (4)
Brazil	1 (2)
China	1 (2)
Germany	1 (2)
Singapore	1 (2)
**Type of publication**	
Empirical	32 (67)
Conceptual	16 (33)
Type of institution referenced regarding institutional COIs*	
Hospital or clinics	28 (58)
Universities or academic institutions	28 (58)
Medical schools	18 (38)
Research Institutes	16 (33)
Other	9 (19)
Unspecified	1 (2)
Recipient(s) of direct financial benefits*	
Researchers & officials	31 (74)
Universities /academic institutions	25 (60)
Hospitals and clinics	25 (60)
Health professionals	21 (44)
Research institutes (not specified)	17 (35)
Medical schools	17 (35)
Other institutions/organisations	7 (15)
Industry involvement*	
Health-related industries (pharmaceutical, biotech, medical device)	38 (79)
Other sectors (food, tobacco, tech, financial services, education)	11 (23)
Unspecified which industry is involved	5 (10)
Research funders (funders, donors, grant, philanthropy)	3 (6)
Healthcare sector	2 (4)
No industry mentioned	1 (2)

* multiple categories per publication possible

The most recent publication in our sample was published in 2022 [28], the earliest was from 1998 [39].

The included literature on institutional COIs is predominantly North American (88%; *n* = 42), with most publications from the United States. Two-thirds of the publications (67%) are empirical, although few employ study designs other than case presentations. The remaining 33% are conceptual. Hospitals or clinics and universities/academic institutions are equally the most referenced institutions regarding institutional COIs (each 58%). The “other institutions” category (15%) includes government and regulatory bodies, internal review boards, and medical societies. When examining the recipients of direct financial benefits, researchers and officials are most frequently mentioned (74%), followed by universities/academic institutions (60%) and hospitals/clinics (60%). Notably, most publications did not distinguish between individual and institutional gains within hospitals and clinics.

The included publications identified health-related industries—such as pharmaceutical, biotechnology, and medical device companies—as the primary source of institutional COIs (79%). Universities and academic institutions were most frequently described as the institutions receiving these industry-derived benefits; however, the distribution of benefits did not differ markedly across the various institution types (see Table 2). Other sectors (e.g., food, tobacco, tech companies) were mentioned in 23% of publications, while research funders without economic interests and the healthcare sector itself were rarely cited.

**Table 2:** Relationships Between the Mentioned Institutions and Involved Industry

Industry Institution	Health-related industries	Healthcare sector	No industry	Other sectors	Research Funders
Hospital or clinics	22	2	1	9	2
Medical Schools	13	1	0	6	1
Other	5	0	0	3	1
Research Institutes	14	1	1	3	1
Universities or academic institutions	23	2	0	8	2
Unspecified	1	0	0	0	0

### Types of COIs

**Fig. 2:**
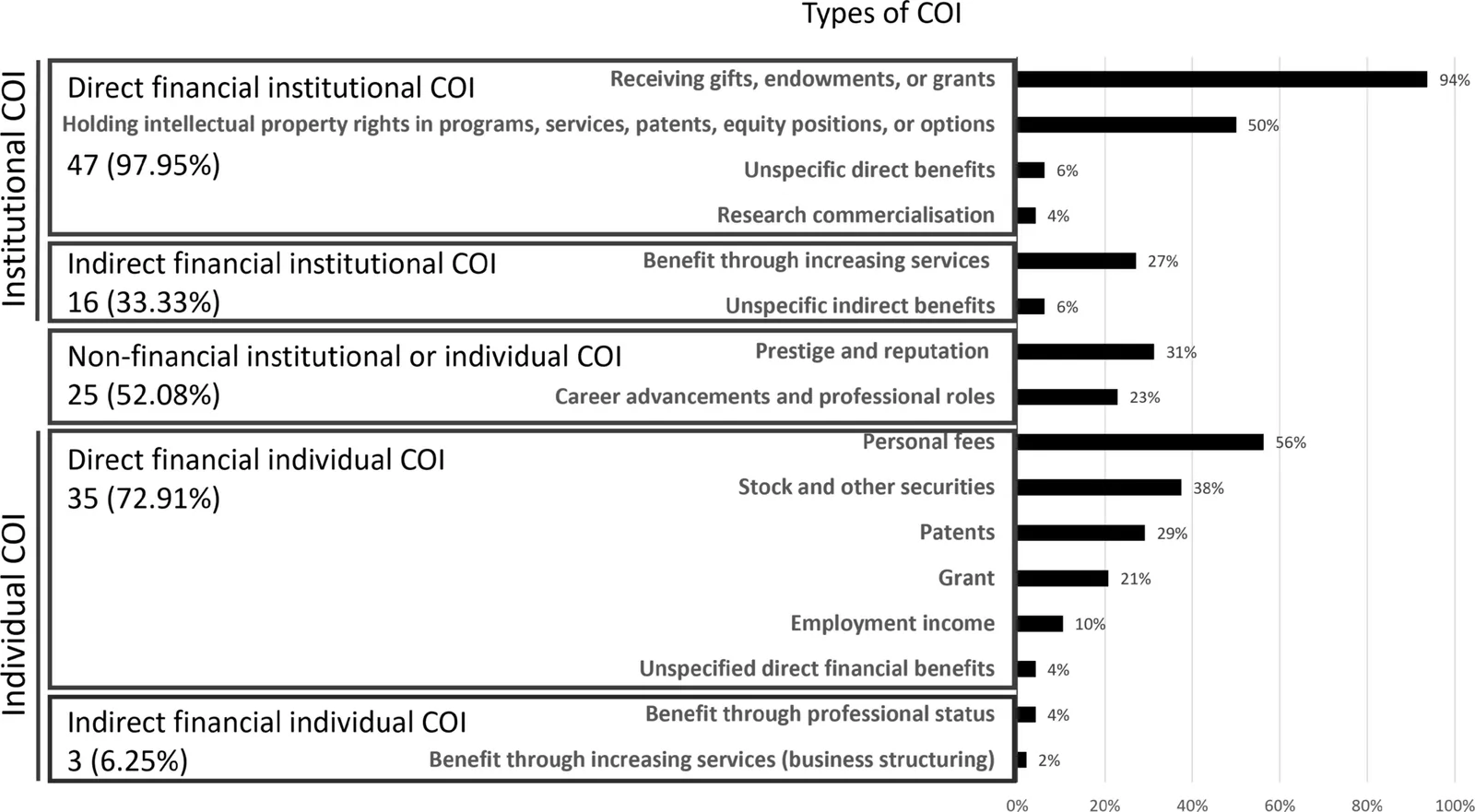
Types of COIs Described in the included Publications. Note: On the left side, the main categories and total number of publications describing the category (%) are indicated. Subcategories were derived from the framework and supplemented thematically. Bar percentages represent % of all 48 included publications. COI = Conflict of Interest. Multiple categories per publication possible

Figure 2 provides an overview of the distribution of types of COIs emerging from the included publications. In general, direct financial COIs were represented in all included publications. Indirect financial (35%; *n* = 17/48) and non-financial COIs (52%; n = 25/48) were much less frequently presented. Table 3 illustrated examples for the five COIs categories derived from the included publications.

**Table 3:** Examples for the different types of COIs

Type of COI	Examples
**Direct financial institutional COIs**	The prominent case of Jesse Gelsinger involved direct financial institutional and individual COIs. The University of Pennsylvania, the principal investigator and inventor (Dr James Wilson), and the commercial sponsor (Biogen) were financially intertwined through shared ownership of a previously funded company (Genovo). This company funded a newly formed institute at the University of Pennsylvania, and this institute directly financed the clinical trial. Consequently, the university and company both stood to benefit financially from the trial’s success. Wilson held multiple roles—university professor, company consultant, and trial co-investigator. These financial COIs may have compromised transparency and patient safety, yet they were not disclosed during the informed consent process. This prompted criticism when analysing the events surrounding Jesse Gelsinger’s death caused by the experimental gene therapy e.g. [5, 10, 30]. The Olivieri case demonstrated how financial ties between the University of Toronto and the drug company Apotex generated COIs. Dr Nancy Olivieri, the lead investigator, tried to warn about major safety risks in a drug trial. Instead of supporting her, the university removed her from her position. Concurrently, a university director assisted Apotex in lobbying against new drug patent regulations, that could have jeopardised funding for the university’s new medical centre, which Apotex had been supporting [30, 40, 60]. This case illustrates how institutional financial interests can override patient safety and scientific integrity.
**Indirect financial institutional COIs**	Economic pressure on medical schools to offer good physical laboratory infrastructure may influence their decision to allow clinical trials with minor relevance to the public population in order to receive funding for these laboratories and thereby attract researchers [45]. In the case of He Jiankui’s Genome-edited babies, the university (Southern University of Science and Technology) recruited He as an acknowledged, rising science star in China despite concerns about ethical violations, because his research promised to raise the institution’s prestige (e.g. no COI policy) [7], that eventually might turn into economic profits.
**Direct financial individual COIs**	A cross-sectional study conducted in 2013 showed that professors or other senior academic officials received substantial monetary fees and stock shares for serving as directors of US healthcare companies, while their professional duty was to conduct unbiased research regardless of profit and medical training [25].
**Indirect financial individual COIs**	Pizzo and two other deans of three research-intensive medical schools reported that pharmaceutical companies sponsored institutional funds or individual faculty members’ research, that in turn supported career development of PhDs or postdoc fellows [58]. Consequently, individuals benefited from their increased professional status.
**Non-financial COIs **	Institutions punished whistleblowers who reported ethical misbehaviour of their own institutions or they avoided thorough investigations of accused misbehaviour of their faculty members to protect their reputation [60]. For example, Dr Robert Sprague alerted the National Institute of Mental Health (NIMH), which funded his grant, about his suspicion that his grant collaborator, Dr Stephen Breuning, had falsified research. The NIMH contacted the University of Pittsburgh, where Dr Breuning was employed then. The university found no serious fault with Dr Breuning’s activities after a few meetings, without investigating his work in Pittsburgh and instead focusing on Dr Breunings previous research at another institution. Three years later, the NIMH condemned Dr Breuning in his final publication for engaging “in serious scientific misconduct”. Dr Sprague, the whistleblower, was also condemned for his “failure to adequately oversee the subcontract”. Consequently, Dr Sprague lost his grant after 17 years of continuous funding even though he had been the first to raise concerns and had insisted that the NIMH investigated Breuning's research [60, 66]. A physician’s designated leadership role in a professional society (career advancement) made him influence the chosen outcomes of a systematic review so that the results aligned with the society’s stated view. No financial ties or strong personal beliefs were involved, but his relation to the society and his own career-advancement goals affected research objectivity [64].

#### Direct financial institutional COIs

Almost all publications cited direct financial institutional COIs (98%; n = 47/48, Additional File 4), which reflected the overall emphasis on financial COIs in publications in general. Most of these COIs originated from receiving gifts, endowments, or grants (94%; n = 45/48). The included publications described compensation for trials, sponsorship of university chairs, conferences, advanced training fees, and departmental/university scholarships as common forms of financial support (see Table 3). Half of the sample reported financial benefits from intellectual property rights in programmes, services, patents, equities, or stock options (50%; n = 24/48). Institutions received, for example, royalties for patents or dividends of shares. A smaller proportion concerned unspecified direct benefits (6%; n = 3/48) and the commercialisation of research (4%; n = 4/48), for example, when universities and start-ups had close ties [12].

#### Indirect financial institutional COIs

One third of publications (33%; n = 16/48, Additional File 4) reported indirect financial institutional COIs. Benefits through increased services formed the main category (27%; n = 13/48), e.g. placing advertisements, partnering with other institutions, mutual referral arrangements, or attracting more applicants to medical schools (see Table 3). A further 6% (n = 3/48) of publications addressed indirect institutional benefits, that were not specified in greater detail.

#### Direct financial individual COIs

We found that direct financial individual COIs were reported in 73% (n = 35/48) of the publications (Additional File 4). More than half of the publications listed personal fees (56%; n = 27/48), while stock and other securities were identified in 38% (n = 18/48) of publications (see Table 3). Patents accounted for 29% (n = 14/48) and grants for 21% (n = 10/48) of the reported individual COIs. The least-frequent categories were employment income from industries and donors (10%; n = 5/48) and other direct financial benefits (4%; n = 2/48), which the publications did not elaborate further.

Thus, the categories reported for institutional and individual financial COIs (grants, stocks, intellectual property/patents) were similar, but their frequency differed, suggesting that different relative importance was ascribed to these categories.

#### Indirect financial individual COIs

We identified indirect financial individual COIs in 6% (n = 3/48) of the included publications (Additional File 4). Two subcategories were derived: benefits through professional status (4%; n = 2/48) and increased services resulting from improved business structuring (2%; n = 1/48).

The publications reported less frequently on individual indirect COIs compared to institutional-level indirect financial COIs, and both levels comprised different subcategories.

#### Non-financial institutional or individual COIs

Because many of the included publications lacked sufficient detail to determine whether non-financial COIs applied to the institution or to an individual, we combined individual and institutional non-financial COIs into a single category. Overall, 52% (n = 25/48) of the publications reported on non-financial COIs (Additional File 4), of which prestige and reputation constituted the largest sub-category (33%; n = 15/48). A further 23% (n = 11/48) described non‑financial COIs related to career advancement and professional roles, encompassing individual gains such as leadership positions and networking, as well as institutional impacts like brain drain.

### Possible consequences of institutional COIs

All publications shared their view on possible (negative) consequences associated with COIs (100%, n = 48/48, Additional file 4). The two major problematic areas presented were “bias and distortion in the process of research” (94%; n = 45/48) and causing “harm to the scientific integrity” (77%; n = 37/48). In the first category, COIs could, for instance, influence the design, objectivity, judgement, the “spin” of results, and the consideration of risks and ethics. The second category included threats to research integrity, risks of legal negligence, and hampering other researchers’ work. For example, industry-sponsored clinical trials created financial incentives for physicians and institutions to enrol patients as study subjects, yet, taking part in the trial may offer little additional benefit to the patients, thereby representing a threat to scientific integrity [30]. Conversely, individual researchers’, educators’, and clinicians’ scientific integrity could be (unfairly) questioned if they were affiliated to an institution which improperly managed COIs even if they had not been involved in the criticised issue [5]. Less frequently cited consequences were “patient safety, public trust, and disadvantages for patients” (17%; n = 8/48), “structural, institutional, or legal weakness” (10%; n = 5/48), and “career-related and reputation-driven conflicts” (4%; n = 2/48).

### Recommendations

Almost all publications (94%; n = 45/48, Additional File 4) provided recommendations on how to mitigate the consequences of COIs or to even avoid COIs. The most frequent recommendation was the application and development of “institutional policies and oversight mechanisms” (77%; n = 37/48). Sixty percent advocated for “financial transparency and restrictions of accepting financial flows”. Other suggested strategies included “legal, ethical and policy development” (31%; n = 15/48) and “cultural and structural change in institutions” (25%; n = 12/48), which encompassed the internal improvements of regulations and the review board as well as a change of culture within the institutions regarding ethics. Moreover, 10% (n = 5/48) of publications recommended “data, transparency, and public engagement” comprising (public) disclosure of COI-related data. Most publications made general, rather vague suggestions on how to manage COIs and did not specify how they could be implemented.

### Additional COI frameworks

Three included publications (6%) reported alternative frameworks and models of COIs or related aspects that could refine the primary framework used for this study [4]. These publications included (i) special attention to the protection of trial participants and involvement of their family members [27], (ii) a different categorisation of relationships between institutions/researchers and industry [35], and (iii) three categories of responses to COIs (disclosure, management, prohibition) [48].

## Discussion

This scoping review of 48 publications provides an overview of COIs arising from institutional affiliation with a healthcare provider in human health-related research involving humans.

Interest in institutional COIs persisted since 2000, but the search did not retrieve any publication from the most recent years (2023 until mid-2024). All included publications agreed that institutional COIs are critical and require addressing. The literature acknowledges that managing institutional COIs may be more difficult than managing individual ones because identifying relevant institutional interests and conflicts within large institutions is challenging, as no individual can be aware of all such interests [5]. Moreover, individual and institutional COIs were most often discussed together and were difficult to disentangle analytically in real life.

The review found that the included publications predominantly examined the situation in North America, indicating a strong geographical concentration, while the only publication from Europe dated back to 1998 [39]. This pattern partially reflects the generally high volume of health-related research originating in the United Stated (cf. [67]), but the degree of overrepresentation observed here seems to exceed typical publication patterns. A similar North-American focus appeared in another scoping review not limited to institutional COIs but that included any ties between industry and a party or activity in the healthcare ecosystem, although studies from 37 countries were included [68]. On the one hand, it one can hypothesise this geographical focus on COIs originated from different healthcare systems; on the other hand, Canada and the US have very different healthcare systems with the latter being much more market-driven. Further research should investigate the reasons for the geographical concentration. In conclusion, there is no reason to assume that COIs are more prevalent to the observed degree in North America, which should be borne in mind to close the knowledge gap about COIs in other parts of the world.

Although most publications incorporated an empirical element, they were often confined to case presentations. We need more interviews, surveys, or systematic analyses of business data to strengthen the empirical evidence on how and to what extent institutional COIs induce bias and reduce the quality of patient care [25, 31, 33]. The same applies to studies including the stance of patients and the public. These perspectives should be taken into consideration when designing new studies for other countries.

In general, direct financial COIs constituted the majority of COIs while indirect financial or non-financial COIs received less attention. There may be several reasons, first of all, direct financial benefits have long been the focus of research on COIs [4], are more obvious, and easier to identify. In addition, at the individual level, there has been a debate whether one should be equally concerned about financial and non-financial COIs. Some researchers have argued that personal interests, which are often non-financial, should not constitute a COI because researchers cannot possibly separate themselves from their beliefs, experiences and professional training [69]. At the institutional level, we believe the distinction between institutional (direct and indirect) financial and non-financial COIs is very narrow, as non-financial institutional COIs can, in the long run, evolve into financial COIs. For example, an institution focussing on a specific topic of research represents a non-financial institutional COI [4]. When researchers working at the institution publish more work on that chosen topic, the institution gains visibility and recognition as an expert in the field. Over time, this reputation can be used to argue that the research area should be prioritised, which in turn can create external funding calls, which the institution aims to attract. Thus, the institution aims at financially benefiting from setting a research focus. The same applies to enhanced prestige or reputation, which can create increased financial benefit. The assumption that institutional financial and non-financial COIs are closely related aligns with a recently published scoping review of non-financial COIs including 190 articles, out of which 36 identified (also) institutional COIs [70]. The authors reported that, “at the institutional level, non-financial interests were seen to be entwined with financial interests”, when for example, the use of novel technologies increased the reputation of an institution (non-financial benefit), thereby increasing patient volume (indirect financial benefit) [70]. In practice, it may be difficult to distinguish between financial and non-financial benefits at the institutional level, and the limited detail provided in the included literature did not allow us to differentiate non-financial COIs at the individual versus institutional level. Nevertheless, the proposed framework [4] remains theoretically useful for increasing awareness of the different types of COIs. Our application of the framework to the literature will be helpful to develop and refine the framework. The authors suggest further refinement of the category naming within the framework. First, it should be made more explicit that the framework includes non-financial COIs of individuals (currently termed “personal” and “intellectual”), consistent with the existing literature [70, 71]. Second, financial benefits that are not direct, could be named indirect financial benefits (currently termed “professional status” (for individuals) and “increasing services provided by institution” (for institutions)).

The definition of institutional COIs employed in this scoping review [4] differs slightly from the definition presented by the Institute of Medicine [5], specifically regarding whether it is the institution’s or the individual’s duty that is influenced by the institution’s benefits. We applied this definition because, in our view, individuals, not the institution itself, carry out research and patient care.

### Management of institutional COIs

To ensure health-related research involving humans is scientifically relevant, practically feasible, and ethically conducted, meaningful engagement of relevant interest holders is essential. Such interest holders commonly include patients, members of the public, healthcare professionals, healthcare service providers, and policy makers [72]. Their engagement can contribute to the identification, assessment, and management of institutional COI. Healthcare professionals as members of a research project group will likely be affiliated with an institution providing treatment for the condition studied, and therefore have an institutional COI, which may influence reporting of study results. For example, authors of scientific articles on mammography screening who were directly involved in screening were less likely than other authors to acknowledge overdiagnosis and overtreatment [73]. This means that the management of institutional COIs in health-related research is very important. However, the awareness of indirect financial or non-financial institutional COIs seems to be lower than of direct financial ones: in a secondary analysis of a systematic review of early intervention studies of autistic children, Bottema-Beutel et al. characterised all authors affiliated to a healthcare provider that provides the intervention as a service, as having a COI, but only few of them disclosed this COI in the COI statement [31].

The most frequently suggested action for managing institutional COIs was the implementation of “institutional policies and oversight mechanisms”. However, only a minority of institutions appear to have such policies: in 2014, a survey of the top 100 US research institutions found that only 28 had an institutional COI policy [74], while surveys conducted in Europe in 2018/2019 indicated that COI policies did not address institutional COIs at all [75, 76]. Although a more recent review found that 61% of organisations producing clinical practice guidelines considered institutional interests to be relevant for disclosure [77], these findings suggest that governance of institutional COIs remains underdeveloped. Existing guidance has proposed several mechanisms for managing institutional COIs: The Institute of Medicine and the Association of American Medical Colleges recommended establishing standing institutional COI committees, separate from those addressing individual COIs, to review the financial relationships of institutions and senior officials, assess whether they conflict with the institution’s mission, and evaluate the adequacy of management procedures [5, 24]. Such committees should include members with sufficient expertise and independence and external (public) members where possible. Incorporating COI screening throughout the research approval process using “trigger” questions, for example regarding possible institutional COIs in the proposed study, may help to identify relevant institutional COIs [27]. Identified COIs should be managed through measures such as independent monitoring of clinical trials and clear separation between research and institutional investment activities [27]. COI management decisions should consider the nature of the institutional interest, its relationship to the research, and the level of risk posed to the research and to participants, while the identified institutional COIs should be reported to the Institutional Review Board or equivalent ethics body [24]. While empirical evidence on the effectiveness of these recommendations remains limited, they illustrate that institutional COIs require dedicated governance structures and oversight mechanisms.

Still, awareness of one's susceptibility to bias and COIs among researchers, administrators, and institutional leaders remains important because COIs can only be disclosed and managed when they are recognised. This mirrors the “culture of skepticism” proposed as a strategy for managing non-financial COIs, beyond mere disclosure [71]. A 2.5-h online training intervention on COIs which included interactive breakout sessions was shown to significantly increase knowledge of how to mitigate risks of corporate funding, and this self-reported knowledge persisted until the follow-up after three months [78]. About half of the participants reported application of the knowledge and tools learned within the course, but very few reported that they had taken action since the training [78]. Institutional COIs were not part of that training (personal communication), but future educational interventions on COIs could incorporate them and study long-term (e.g. one year) behavioural change after exposure to training interventions. This could enhance awareness of bias stemming from institutional COIs.

### Strengths and limitations

The strength of this review lies in its systematic approach, consolidating and synthesising research on healthcare provider-associated institutional COIs. While the importance of institutional COIs has been acknowledged, there was no systematic categorisation of the types of institutional COIs previously.

Despite our best efforts, there are several potential limitations in this study. First, we searched only two healthcare-related databases and did not perform citation chasing. Thus, we might have missed relevant literature published in other fields. Second, we only included publications written in English and German, so articles published in other languages which may discuss COIs were not captured. Third, identifying relevant publications proved difficult, as the term “institutional COI” is not always used; consequently, some publications may have elaborated on institutional COIs without naming them as such in their title or the abstract (for example, [73]). Nevertheless, our search strategy focused on studies that mentioned institutional conflicts of interest or relationships with industry in some form in the title or abstract and therefore did not identify such potentially eligible publications referring to payments, funding etc. Fourth, we did not assess whether the described interests qualify as COIs [4] but relied on the description of the included papers. Fifth, individual and institutional COIs were not always clearly distinguishable. When discussing potential consequences and offering recommendations for COI management, the included publications did not consistently indicate whether they referred to institutional or individual COIs. Consequently, we could not achieve a definitive separation of the two COI concepts in this review. At the same time, combining COIs at the individual and institutional level COIs in one scoping reviews makes sense, also because this allows comparing and contrasting financial and non-financial COIs at both levels. Sixth, we did not distinguish whether or not the possible consequences of COIs were empirically supported or simply discussed.

### Future research

Future studies on institutional COIs of healthcare providers should address geographical areas other than North America and employ empirical study designs beyond case presentations. For example, future surveys could elicit researchers’ opinions on implications of institutional COIs. A systematic analysis of research institutions’ financial ties and their association with study outcomes (literature analysis) could allow researchers to empirically determine the nature, impact, and mitigating measures for institutional COIs. Future evidence syntheses on institutional COIs could extend our focus on healthcare providers involved in health-related research involving humans and should employ more sensitive search strategies that identify publications on institutional COIs without naming them as such. More research is needed to understand the extent and impact of indirect financial and non-financial COIs and how financial and non-financial institutional COIs relate. Moreover, training programmes on COIs that incorporate institutional COIs should be developed and evaluated for their ability to enhance awareness and induce behavioural change.

## Conclusions

This scoping review summarises how COIs arising from institutional affiliation with a healthcare provider are described in health-related research involving humans. The publications predominantly describe the situation in North America and COIs mostly stem from relationships with health-related industries. Direct financial COIs dominate the literature, while indirect or non-financial COIs were less often investigated. Individual and institutional COIs were most often discussed together. Authors always acknowledged possible negative consequences of institutional COIs and most often suggested COI policies to manage them, yet most institutions lack policies which cover institutional COIs. Providing training programmes to raise awareness of institutional COIs may reduce bias and enhance research integrity.

## Supplementary Information

Additional file 1: PRISMA-Scr checklist.

Additional file 2: Search strategies.

Additional file 3: Data extraction file of individual studies.

Additional file 4: Study characteristics.

## Data Availability

All data generated or analysed during this study are included in this published article and its supplementary information files.

## Abbreviations

AI: Artificial intelligence
COI: Conflict of interest
PRESS: Peer Review of Electronic Search Strategies
PRISMA-ScR: Preferred Reporting Items for Systematic review and Meta-Analysis extension for Scoping Reviews PRISMA
