# Supplementary material for: Institutional conflicts of interest in health-related research — a scoping review
Source: Res Integr Peer Rev. 2026 Sep 16;11:52. doi: 10.1186/s41073-026-00251-w (PMC13584590; doi:10.1186/s41073-026-00251-w)
Supplement: Supplementary file 2 [file 41073_2026_251_MOESM2_ESM.pdf]

1 Additional file 1: Search Strategies

Haarmann A et al: Institutional Conflicts of Interest in Health-related Research — A Scoping Review.

Search Strategy for PubMed

"icoi"[Title/Abstract] OR "icois"[Title/Abstract]

OR

(("institutional conflict of interest"[tiab:~2] OR "institutional conflict of interests"[tiab:~2] OR "institutional conflicts of interest"[tiab:~2] OR "institutional conflicts of interests"[tiab:~2] OR "institutional conflict of duty"[tiab:~2] OR "institutional relationship with industry"[tiab:~2] OR "institutional coi"[tiab:~2] OR "institutional cois"[tiab:~2]

OR

"institutions conflict of interest"[tiab:~2] OR "institutions conflict of interests"[tiab:~2] OR "institutions conflicts of interest"[tiab:~2] OR "institutions conflicts of interests"[tiab:~2] OR "institutions conflict of duty"[tiab:~2] OR "institutions relationship with industry"[tiab:~2] OR "institutions coi"[tiab:~2] OR "institutions cois"[tiab:~2]

OR

"institution conflict of interest"[tiab:~2] OR "institution conflict of interests"[tiab:~2] OR "institution conflicts of interest"[tiab:~2] OR "institution conflicts of interests"[tiab:~2] OR "institution conflict of duty"[tiab:~2] OR "institution relationship with industry"[tiab:~2] OR "institution coi"[tiab:~2] OR "institution cois"[tiab:~2]

OR

"institutes conflict of interest"[tiab:~2] OR "institutes conflict of interests"[tiab:~2] OR "institutes conflicts of interest"[tiab:~2] OR "institutes conflicts of interests"[tiab:~2] OR "institutes conflict of

duty"[tiab:~2] OR "institutes relationship with industry"[tiab:~2] OR "institutes coi"[tiab:~2] OR "institutes cois"[tiab:~2]

OR

"institute conflict of interest"[tiab:~2] OR "institute conflict of interests"[tiab:~2] OR "institute conflicts of interest"[tiab:~2] OR "institute conflicts of interests"[tiab:~2] OR "institute conflict of duty"[tiab:~2] OR "institute relationship with industry"[tiab:~2] OR "institute coi"[tiab:~2] OR "institute cois"[tiab:~2]

OR

("institutional"[Title/Abstract] OR "institutions"[Title/Abstract] OR "institution"[Title/Abstract] OR "institutes"[Title/Abstract] OR "institute"[Title/Abstract]) AND "conflict of interest"[MeSH Terms]))

Date of search: 31 July 2024

Hits: 801

Search Strategy for Embase

('icoi':ti,ab,kw OR 'icois':ti,ab,kw OR ((institutional NEAR/2 ('conflict of interest' OR 'conflict of interests' OR 'conflicts of interest' OR 'conflicts of interests' OR 'conflict of duty' OR 'relationship with industry' OR coi OR cois)):ti,ab,kw) OR ((institutions NEAR/2 ('conflict of interest' OR 'conflict of interests' OR 'conflicts of interest' OR 'conflicts of interests' OR 'conflict of duty' OR 'relationship with industry' OR coi OR cois)):ti,ab,kw) OR ((institution NEAR/2 ('conflict of interest' OR 'conflict of interests' OR 'conflicts of interest' OR 'conflicts of interests' OR 'conflict of duty' OR 'relationship with industry' OR coi OR cois)):ti,ab,kw) OR ((institutes NEAR/2 ('conflict of interest' OR 'conflict of interests' OR 'conflicts of interest' OR 'conflicts of interests' OR 'conflict of duty' OR 'relationship with industry' OR coi OR cois)):ti,ab,kw) OR (('institutional':ti,ab,kw OR 'institutions':ti,ab,kw OR 'institution':ti,ab,kw

OR 'institutes':ti,ab,kw OR 'institute':ti,ab,kw) AND 'conflict of interest'/exp)) AND [embase]/lim NOT [medline]/lim NOT [pubmed-not-medline]/lim NOT [conference abstract]/lim

Date of search: 31 July 2024

Hits: 115

Search Strategy for GoogleScholar

intitle:"institutional conflict of interest"|intitle:"institutional conflicts of interest"

Date of search: 31 July 2024

Hits: 70
