# Supplementary material for: Institutional conflicts of interest in health-related research — a scoping review
Source: Res Integr Peer Rev. 2026 Sep 16;11:52. doi: 10.1186/s41073-026-00251-w (PMC13584590; doi:10.1186/s41073-026-00251-w)
Supplement: Supplementary file 3 [file 41073_2026_251_MOESM4_ESM.pdf]

Additional File 2: Study characteristics

Haarmann A et al: Institutional Conflicts of Interest in Health-related Research — A Scoping Review.

Characteristics	References	Full dataset (N=48) Frequency (%)
**Year of publication**		
Before 2000	(39)	1 (2.08)
2000-2004	(24, 26, 27, 32, 35, 36, 41, 50, 57, 60, 62, 65)	12 (25.00)
2005-2009	(5, 9, 34, 37, 40, 42, 45, 47, 56)	9 (18.75)
2010-2014	(30, 43, 44, 46, 49, 51, 59, 63, 64)	9 (18.75)
2015-2019	(12, 25, 29, 38, 48, 53-55, 58)	9 (18.75)
2020-2022	(7, 10, 11, 28, 31, 33, 52, 61)	8 (16.67)
**Country, region***		
USA	(5, 9, 11, 12, 24-27, 29, 30, 32-38, 42, 43, 46, 47, 49-51, 53-60, 62, 64, 65)	35 (72.92)
Canada	(26, 30, 40, 41, 44, 60, 61)	7 (14.58)
Global	(28, 31, 41, 45)	4 (8.33)
Australia	(40, 48)	2 (4.17)
Japan	(10, 63)	2 (4.17)
Brazil	(45)	1 (2.08)
China	(7)	1 (2.08)
Germany	(39)	1 (2.08)
Singapore	(52)	1 (2.08)
**Type of Study**		
Empirical	(7, 9-11, 25, 26, 28-31, 33, 35-38, 40-42, 44, 46-50, 53, 54, 59-62, 65)	32 (66.7)
Conceptual		16 (33.3)
**Type of institution referenced regarding institutional COI***		
Hospital or clinics	(7, 9, 10, 12, 24-31, 33, 35, 38-40, 45, 48, 51-53, 55-57, 60, 61, 65)	28 (58.33)
Universities or academic	(7, 10, 24-36, 39, 41-44, 47, 48, 58-60, 63, 65)	28 (58.33)
Medical schools	(10, 12, 25, 27, 28, 34, 35, 37, 42, 43, 45, 49, 50, 54, 56, 62, 65)	18 (37.50)
Research Institutes	(5, 11, 24, 25, 27, 30, 31, 34, 35, 39-41, 45, 46, 63)	16 (33.33)
Other	(11, 27, 28, 33, 39, 42, 46, 57, 63)	9 (18.75)
Unspecified	(64)	1 (2.06)
**Recipient(s) of direct financial benefits***		
Researchers & officials	(5, 9, 11, 24-28, 30, 33-36, 38-42, 44-47, 50-52, 55, 62, 64)	31 (73.81)
Universities /academic	(5, 7, 10, 24-27, 29, 30, 33, 35, 39, 41, 43, 44, 47, 48, 52, 55, 58-60, 63-65)	25 (59.52)
Hospitals and clinics	(7, 9-12, 24-26, 28, 29, 31, 33, 35, 38, 40, 45, 48, 51, 53, 55-57, 60, 61, 65)	25 (59.52)
Health professionals	(7, 9, 28-30, 36, 38-41, 43, 45-47, 49-51, 57-59)	21 (43.75)
Research institutes (not specified)	(5, 11, 24, 25, 27, 31, 33-35, 37, 38, 41, 45, 46, 62-64)	17 (35.42)
Medical schools	(10, 12, 25, 28, 30, 35, 42, 43, 45, 49, 50, 54, 56, 58, 59, 62, 63, 65)	17 (35.42)
Other institutions/organisations	(24, 27, 45, 46, 48, 53, 54)	7 (14.58)

Characteristics	References	Full dataset (N=48) Frequency (%)
**Industry involved***		
Health-related industries (pharmaceutical, biotech, medical -device)	(5, 7, 9, 10, 12, 24-27, 29-31, 34-36, 39-41, 43-51, 53-55, 57-64)	38 (79.17)
Other sectors (food, tobacco, tech, financial services, education)	(12, 24, 27, 28, 31, 33, 43, 44, 52, 56, 65)	11 (22.92)
Unspecified, which industry is involved	(11, 32, 37, 42, 48)	5 (10.42)
Research funders (funders, donors, grant, philanthropy)	(31, 42, 52)	3 (6.25)
Healthcare sector	(25, 29)	2 (4.17)
No industry mentioned	(38)	1 (2.08)
**Institutional COI text focus**		
Institutional COI exclusively	(5, 10-12, 34-37, 39, 40, 43, 44, 46, 52-56, 60-62, 65)	22 (45.83)
Institutional COI is the main focus & individual COI subordinate role	(24, 26-28, 30, 32, 38, 41, 42, 51)	10 (20.83)
Mentions both institutional and individual COI to about the same extent	(7, 9, 25, 33, 47, 49, 50, 58, 64)	9 (18.75)
Individual COI is the main focus & institutional COI subordinate role	(29, 31, 45, 48, 63)	5 (10.42)
Individual COI is the main focus	(59)	1 (2.08)
Primary focus on business, legal and regulatory aspects of physician joint ventures is the main focus & institutional COI subordinate role	(56)	1 (2.08)
**Direct financial institutional COI***	**(5, 7, 9-12, 24, 26-65)**	**47 (97.95)**
Receiving gifts, endowments, or grants	(5, 7, 9-12, 24, 26-35, 37-48, 50-65)	45 (93.75)
Holding intellectual property rights of programmes, services, patents, equity positions, or options	(7, 9, 12, 26, 27, 31, 32, 34, 35, 39, 41, 42, 47, 50, 51, 54-56, 58, 59, 64, 65)	24 (50.0)
Unspecified direct benefits	(24, 40, 49)	3 (6.25)
Research commercialisation	(5, 12)	2 (4.17)
**Indirect financial institutional COI***	**(7, 10, 12, 24, 27, 28, 31, 33, 34, 41, 43-45, 49, 55, 58)**	**16 (33.33)**
Benefit through increasing services	(7, 10, 12, 31, 33, 34, 41, 43-45, 49, 55, 58)	13 (27.08)
Unspecified indirect benefits	(24, 26, 28)	3 (6.12)
**Direct financial individual COI***	**(7, 9, 12, 24-32, 34-37, 39-41, 46-48, 50-52, 54-60, 63-65)**	**35 (72.91)**
Personal fees	(12, 25, 27-31, 35, 37, 39, 41, 46-48, 50-52, 54, 55, 57-59, 63-65)	27 (56.25)
Stock and other securities	(7, 9, 12, 24, 25, 27, 29, 31, 32, 34, 36, 37, 39, 47, 51, 57, 59, 65)	18 (37.50)
Patents	(9, 12, 29, 31, 32, 34, 35, 41, 47, 50, 51, 55, 59, 64)	14 (29.17)
Grant	(7, 12, 27, 28, 31, 40, 46, 51, 55, 60)	10 (20.83)
Employment income	(9, 27, 48, 50, 54)	5 (10.42)

Characteristics	References	Full dataset (N=48) Frequency (%)
Unspecified direct financial benefits	(26, 56)	2 (4.17)
**Indirect financial individual COI***	**(7, 57, 58)**	**3 (6.25)**
Benefit through professional status	(7, 58)	2 (4.17)
Benefit through increasing services (business structuring)	(57)	1 (2.08)
**Non-financial institutional or individual COI***	**(7, 11, 12, 27, 28, 31, 34, 35, 41, 43, 45, 48, 51-56, 58-61, 63, 64)**	**25 (52.08)**
Prestige and reputation	(7, 12, 27, 28, 32, 41, 43, 48, 51, 52, 54, 55, 60, 61)	15 (31.25)
Career advancements and professional roles	(11, 31, 34, 35, 45, 53, 54, 56, 59, 63)	11 (22.92)
**Possible consequences of institutional COIs***	**(5, 7, 9-12, 24-65)**	**48 (100%)**
Bias & distortion in research process	(5, 7, 9-12, 27-33, 35-65)	45 (93.8)
Harm to scientific integrity	(5, 7, 10-12, 24-27, 31-33, 35, 37, 38, 40-45, 47-49, 51, 52, 54-56, 58-65)	37 (77.08)
Patient safety, public trust & disadvantages for patients	(9, 26, 28, 38, 46, 53, 54, 57)	8 (16.67)
Structural, institutional, or legal weaknesses	(34, 39, 40, 42, 64)	5 (10.42)
Career-related & reputation-driven conflicts	(39, 64)	2 (4.17)
**Institutional COI management recommendations***	**(5, 7, 9, 10, 12, 24-34, 36, 38-65)**	**45 (93.8)**
Institutional policies & oversight mechanisms	(5, 7, 9, 10, 12, 24-31, 34, 36, 38, 39, 41-45, 49, 51-60, 62, 63, 65)	37 (77.08)
Financial transparency & restrictions of financial flows	(5, 7, 9, 10, 24, 25, 27, 28, 30-34, 36, 39, 42, 43, 45-48, 50, 51, 56-59, 62, 65)	29 (60.42)
Legal, ethical & policy development	(7, 10, 12, 29-31, 33, 40, 43, 44, 50, 51, 61-63)	15 (31.25)
Cultural and structural change in institutions	(7, 24, 28, 36, 43-45, 49, 57, 60, 63, 64)	12 (25.0)
Data, transparency & public engagement	(33, 34, 43, 56, 62)	5 (10.42)

Note: Multiple entries possible; base for the calculation of percentages is the number of reports

References:

5. Institute of Medicine. Chapter 8 Institutional Conflicts of Interest. In: Lo B, Field MJ, editors. Conflict of Interest in Medical Research, Education, and Practice. Washington, DC: The National Academies Press; 2009.
7. Nie JB, Xie G, Chen H, Cong Y. Conflict of Interest in Scientific Research in China: A Socio-ethical Analysis of He Jiankui's Human Genome-editing Experiment. J Bioeth Inq. 2020;17(2):191–201.
9. Camilleri M, Gamble GL, Kopecky SL, Wood MB, Hockema ML. Principles and process in the development of the Mayo Clinic's individual and institutional conflict of interest policy. Mayo Clin Proc. 2005;80(10):1340–6.
10. Ozaki A, Murayama A, Harada K, Saito H, Sawano T, Tanimoto T, et al. How Do Institutional Conflicts of Interest Between Pharmaceutical Companies and the Healthcare Sector Become Corrupt?

A Case Study of Scholarship Donations Between Department of Clinical Anesthesiology, Mie University, and Ono Pharmaceutical in Japan. Front Public Health. 2021;9:762637.
11. Varady NH, Worsham CM, Jena AB. Analysis of institutional conflict of interest and bias in research findings and reporting. Healthcare. 2021;9(1):100515.
12. Resnik DB. Institutional Conflicts of Interest in Academic Research. Sci Eng Ethics. 2019;25(6):1661–9.
24. AAMC Task Force on Financial Conflicts of Interests in Clinical Research. Protecting Subjects, Preserving Trust, Promoting Progress II: Principles and Recommendations for Oversight of an Institution's Financial Interests in Human Subjects Research. Academic Medicine, Management Series: Manageing the Research Enterprise. 2003:87–93.
25. Anderson TS, Good CB, Gellad WF. Prevalence and compensation of academic leaders, professors, and trustees on publicly traded US healthcare company boards of directors: cross sectional study. BMJ (Clinical research). 2015;351:h4826.
26. Barnes M, Florencio P. Investigator, IRB and Institutional Financial Conflicts of Interest in Human-Subjects Research: Past, Present and Future. Seton Hall law review. 2003;32:525–60.
27. Barnes M, Florencio PS. Financial conflicts of interest in human subjects research: the problem of institutional conflicts. J Law Med Ethics. 2002;30(3):390–402.
28. Becker GE, Constance C, Tuan TN, Jennifer C, Paul Z, Roger M. Babies before business: protecting the integrity of health professionals from institutional conflict of interest. BMJ Global Health. 2022;7(8):e009640.
29. Bennett J. Institutional Conflict of Interest. JAMA Ophthalmology. 2016;134(11):1334–5.
30. Bienkowski R. Institutional Financial Conflicts of Interest in Clinical Research. 2011.
31. Bottema-Beutel K, Crowley S, Sandbank M, Woynaroski TG. Research Review: Conflicts of Interest (COIs) in autism early intervention research - a meta-analysis of COI influences on intervention effects. J Child Psychol Psychiatry. 2021;62(1):5–15.
32. Bradley G. Institutional Conflict Of Interest. The Journal of Research Administration. 2002;33(2):27–35.
33. Bragg MA, Elbel B, Nestle M. Food Industry Donations to Academic Programs: A Cross-Sectional Examination of the Extent of Publicly Available Data. Int J Environ Res Public Health. 2020;17(5):1624.
34. Broccolo BM, Geetter JS. Today's conflict of interest compliance challenge: how do we balance the commitment to integrity with the demand for innovation? J Health Life Sci Law. 2008;1(4):1,–3–65.
35. Campbell EG, Blumenthal D. Academic industry relationships in biotechnology: a primer on policy and practice. Cloning. 2000;2(3):129–36.
36. Campbell EG, Moy B, Feibelmann S, Weissman JS, Blumenthal D. Institutional academic industry relationship: results of interviews with university leaders. Account Res. 2004;11(2):103–18.
37. Campbell EG, Weissman JS, Ehringhaus S, Rao SR, Moy B, Feibelmann S, et al. Institutional academic industry relationships. JAMA. 2007;298(15):1779–86.
38. Collins ME, Rum S, Wheeler J, Antman K, Brem H, Carrese J, et al. Ethical Issues and Recommendations in Grateful Patient Fundraising and Philanthropy. Academic Medicine. 2018;93:1631–7.
39. Dietel M. Unerlaubte Zuwendungen aus Sicht der universitären Forschung. Zeitschrift für ärztliche Fortbildung und Qualitätssicherung im Gesundheitswesen. 1998;92:620–6.
40. Downie J, Thompson J, Baird P, Dodds S. The Olivieri Case: Lessons for Australasia. Articles, Book Chapters, & Popular Press. 2005.
41. DuVal G. Institutional Conflicts of Interest: Protecting Human Subjects, Scientific Integrity, and Institutional Accountability. Journal of Law, Medicine & Ethics. 2004;32(4):613–25.
42. Ehringhaus SH, Weissman JS, Sears JL, Goold SD, Feibelmann S, Campbell EG. Responses of Medical Schools to Institutional Conflicts of Interest. JAMA. 2008;299(6):665–71.
43. Falit BP, Halperin EC, Loeffler JS. Green eggs and ham: Strategies to address the growing phenomenon of selling a medical school's name. Acad Med. 2014;89(12):1614–6.

44. Gillis MR, McNally ME. The Influence of Industry on Dental Education. Journal of Dental Education. 2010;74(10):1095–105.
45. Greco D, Diniz NM. Chapitre 9. Conflicts of Interest in Research Involving Human Beings. Journal International de Bioéthique. 2008;19(1):143–54.
46. Greenberg RD. Conflicts of Interest: can a physician serve two masters? Clinics in Dermatology. 2012;30(2):160–73.
47. Hampson LA, Agrawal M, Joffe S, Gross CP, Verter J, Emanuel EJ. Patients' views on financial conflicts of interest in cancer research trials. The New England Journal of Medicine. 2006;355(22):2330–7.
48. Jacmon H. Disclosure is Inadequate as a Solution to Managing Conflicts of Interest in Human Research. Bioethical Inquiry. 2018;15(1):71–80.
49. Jones JW, McCullough LB. When is medical industry backing lacking? J Vasc Surg. 2012;55(6):1810–1.
50. Kim SYH, Millard RW, Nisbet P, Cox C, Caine ED. Potential research participants' views regarding researcher and institutional financial conflicts of interest. J Med Ethics. 2004;30(1):73–9.
51. Liang BA, MacKey T. Confronting Conflict: Addressing Institutional Conflicts of Interest in Academic Medical Centers. American Journal of Law & Medicine. 2010;36(1):136–87.
52. Lo YL. Further insights on institutional conflicts of interest in research settings. Proceedings of Singapore Healthcare. 2020;29(1):3–4.
53. McKinney RE, Jr. Institutional Conflict of Interest and the Translational Pathway. JAMA Ophthalmology. 2016;134(5):483–4.
54. Mello MM, Murtagh L, Joffe S, Taylor PL, Greenberg Y, Campbell EG. Beyond financial conflicts of interest: Institutional oversight of faculty consulting agreements at schools of medicine and public health. PLoS One. 2018;13(10):e0230179.
55. Meslin EM, Rager JB, Schwartz PH, Quaid KA, Gaffney MM, Duke J, et al. Benchmarks for ethically credible partnerships between industry and academic health centers: beyond disclosure of financial conflicts of interest. Clin Transl Med. 2015;4(1):36.
56. Miller ED. Creating an institutional conflict-of-interest policy at Johns Hopkins: progress and lessons learned. Cleve Clin J Med. 2007;74 Suppl 2:S70–2; discussion S7–80.
57. Miller JN. Physician joint ventures: new opportunities and risks. J Med Pract Manage. 2004;19(6):292–5.
58. Pizzo PA, Lawley TJ, Rubenstein AH. Role of Leaders in Fostering Meaningful Collaborations Between Academic Medical Centers and Industry While Also Managing Individual and Institutional Conflicts of Interest. JAMA. 2017;317(17):1729–30.
59. Raad R, Appelbaum PS. Relationships between medicine and industry: approaches to the problem of conflicts of interest. Annu Rev Med. 2012;63:465–77.
60. Rhodes R, Strain JJ. Whistleblowing in academic medicine. J Med Ethics. 2004;30(1):35–9.
61. Schafer A. Institutional conflict of interest: attempting to crack the deferiprone mystery. J Med Ethics. 2021;47(8):531–8.
62. Schulman KA, Seils DM, Timbie JW, Sugarman J, Dame LA, Weinfurt KP, et al. A National Survey of Provisions in Clinical-Trial Agreements between Medical Schools and Industry Sponsors. The New England Journal of Medicine. 2002;347(17):1335–41.
63. Shimazawa R, Ikeda M. Conflicts of interest in psychiatry: strategies to cultivate literacy in daily practice. Psychiatry Clin Neurosci. 2014;68(7):489–97.
64. Viswanathan M, Carey TS, Belinson SE, Berliner E, Chang SM, Graham E, et al. A proposed approach may help systematic reviews retain needed expertise while minimizing bias from nonfinancial conflicts of interest. Journal of Clinical Epidemiology. 2014;67(11):1229–38.
65. Wander N, Malone RE. Selling off or selling out? Medical schools and ethical leadership in tobacco stock divestment. Acad Med. 2004;79(11):1017–26.
